# Precision Profiling of Disease Progression in Murine Models of Sepsis and Septic Shock

**DOI:** 10.3390/ijms26209954

**Published:** 2025-10-13

**Authors:** Stewart D. Ramsay, Declan E. Kilgariff, Benjamin J. Young, Mark P. Plummer, Marni A. Nenke, Emily J. Meyer, David J. Torpy, Richard L. Young

**Affiliations:** 1 Intestinal Sensing Group, The University of Adelaide, Adelaide, SA 5005, Australia; stewart.ramsay@adelaide.edu.au (S.D.R.); declan.kilgariff@adelaide.edu.au (D.E.K.); benjamin.j.young@adelaide.edu.au (B.J.Y.); 2 Adelaide Medical School, The University of Adelaide, Adelaide, SA 5005, Australia; mark.plummer@sa.gov.au (M.P.P.); marni.nenke@sa.gov.au (M.A.N.); emily.meyer@sa.gov.au (E.J.M.); david.torpy@sa.gov.au (D.J.T.); 3 Diabetes, Nutrition & Gut Health, Lifelong Health, South Australian Health & Medical Research Institute (SAHMRI), Adelaide, SA 5000, Australia; 4 Intensive Care Unit, Royal Adelaide Hospital, Adelaide, SA 5000, Australia; 5 Endocrine and Metabolic Unit, Royal Adelaide Hospital, Adelaide, SA 5000, Australia; 6 Centre of Research Excellence in Translating Nutritional Science to Good Health, The University of Adelaide, Adelaide, SA 5005, Australia

**Keywords:** sepsis, septic shock, telemetry

## Abstract

Septic shock has an unacceptably high mortality rate and unmet need for new therapeutics. Murine models are crucial for research, yet methodologies often differ. This study characterised standard- and high-grade caecal ligation and puncture (CLP) murine models of septic shock by integrating ultraminiature arterial telemetry with comprehensive plasma biomarker analysis. Standard-grade and high-grade CLP was performed in 8–10 week old, male C57BL/6 mice (n = 98), with a subset implanted with arterial telemetry to monitor real-time circulatory function. Plasma markers of inflammation and organ damage were measured at multiple intervals up to 168 h post-CLP. Standard-grade and high-grade CLP showed distinct progressions; episodes of hypotension began 5–6 h after CLP in 30% of standard-grade and all high-grade CLP mice, with respective 168 h mortality of 40% and 71%. Recurrent episodes of hypotension 5–39 h after CLP were universally lethal. The coincidence of hypotension and elevated plasma lactate defined the onset of septic shock after high-grade CLP, which was always lethal. Inflammatory cytokines and markers of liver, renal, and cardiac damage were markedly elevated to 168 h after high-grade CLP, in contrast to standard-grade CLP, which returned to baseline by 48 h. Elevated plasma IL-6, TNFα, and corticosterone, along with reduced albumin, were significantly correlated with mortality. In conclusion, this research refines murine CLP models by providing a precise, dynamic map of the progression to septic shock. The high-grade CLP model consistently models early and late-stage physiological deterioration and serves as a robust model for evaluating the efficacy of novel therapies aimed at human septic shock.

## 1. Introduction

Sepsis is responsible for 1 in 5 deaths worldwide and is characterised by an extreme physiological response to a pathogenic bacterial, viral, or fungal infection, causing inflammation-mediated organ dysfunction [1,2]. In severe cases, sepsis progresses to septic shock, characterised by a persistent elevation in circulating lactate and pro-inflammatory cytokines [3], and nitric-oxide-driven vasodilation, which leads to hypotension, multi-organ failure [4] and mortality up to 50% [5]. Pro-inflammatory cytokines also activate the hypothalamic–pituitary–adrenal (HPA) axis stress response to increase endogenous glucocorticoids such as cortisol (corticosterone in rodents), leading to vasopressor and anti-inflammatory actions [2,6].

The standard treatment for septic shock within an intensive care unit (ICU) includes source control, antimicrobials, fluid resuscitation, and vasopressor support. Glucocorticoids, such as hydrocortisone, are indicated for refractory septic shock, which has an attributable mortality as high as 50% [5,7,8]. However, glucocorticoid therapy often fails, with systematic and meta-analyses demonstrating limited 28-day survival benefits [9]. Despite the high mortality rate of septic shock and high failure rate of standard treatment, no new therapies have been translated into standard practice in over two decades [10]. The development of any new therapy first requires a suitable, consistent, and reliable preclinical animal model of sepsis and septic shock, with disease progression akin to that observed in humans.

Animal models of sepsis include acute endotoxemia models following lipopolysaccharide (LPS) [11,12] or *Escherichia coli* [13] administration and polymicrobial models following caecal ligation and puncture (CLP) [14]. In general, endotoxemia models demonstrate dose-dependent inflammation, with higher doses leading to higher mortality [15]; however, these models do not recapitulate the hemodynamic changes or multi-organ failure that commonly occur during sepsis in clinical settings [16,17].

CLP is considered the current gold standard [14] for modelling sepsis and its sequelae in small mammals. In rodents, CLP involves ligation and puncture of the caecum to allow bacteria egress to the peritoneal cavity to induce polymicrobial sepsis [14]. The severity of sepsis induced depends on several factors, including the length of the caecum ligated [18] and the puncture size and number [19]. Generally, the larger the ligation and puncture and the increased number of punctures, the higher the mortality [18,20]. CLP-induced sepsis leads to hemodynamic instability [21,22] and multi-organ damage [23], akin to what is observed clinically in sepsis.

While the progression of sepsis in endotoxemia and CLP models has been tracked by measuring changes in circulating levels of cytokines and systemic and organ damage biomarkers, the hemodynamic characteristics of septic shock are rarely tracked. Implantable ultraminiature wireless telemetry offers a contemporary solution in septic shock models, enabling real-time monitoring of heart rate, systolic and diastolic blood pressure, temperature, and activity [24]. These physiological parameters can provide valuable insight into septic shock progression, while minimising experimental interference.

Accordingly, this study utilised ultraminiature wireless telemetry in conjunction with tracking of multiple blood biomarkers to precisely map sepsis onset and progression to septic shock in mice following standard- and high-grade CLP.

## 2. Results

### 2.1. Survival Series

**Figure 1:**
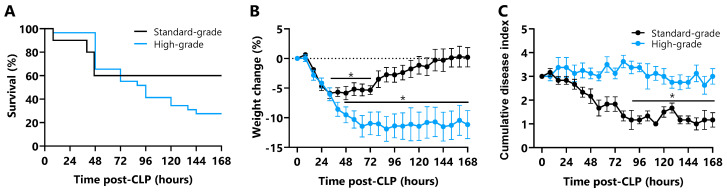
**Survival, weight change, and morbidity of CLP mice.** (**A**) 168 h Kaplan–Meier survival curve of male C57BL/6 mice subject to standard- (n = 10) or high-grade CLP (n = 28). (**B**) Weight change % following standard- and high-grade CLP. (**C**) Cumulative disease index score following standard- and high-grade CLP (high score indicates high morbidity). Data are presented as mean ± SEM; * *p* ≤ 0.05.

The effect of standard- and high-grade CLP on mortality, weight, and CDI score in mice is shown in Figure 1. Standard-grade CLP resulted in a 7-day mortality rate of 40% (4/10) while high-grade CLP resulted in 71% mortality (20/28, *p* ≤ 0.01, Figure 1A). Body weight decreased relative to preoperative weight in all CLP mice; weight was lower between 32 and 72 h in standard-grade CLP mice (5–6%, *p* ≤ 0.05) before a return to preoperative baseline, while weight was lower from 40 h in high-grade CLP mice (9–11%, *p* ≤ 0.001) and did not regain through to 168 h (Figure 1B). Relative to control mice (zero CDI score), CDI scores were elevated in all CLP mice at 6 h and decreased to 60% lower in standard-grade CLP mice from 88 to 168 h (*p* ≤ 0.05); CDI score, however, remained elevated through 168 h in high-grade CLP mice (Figure 1C).

### 2.2. Mean Arterial Pressure (MAP)

**Figure 2:**
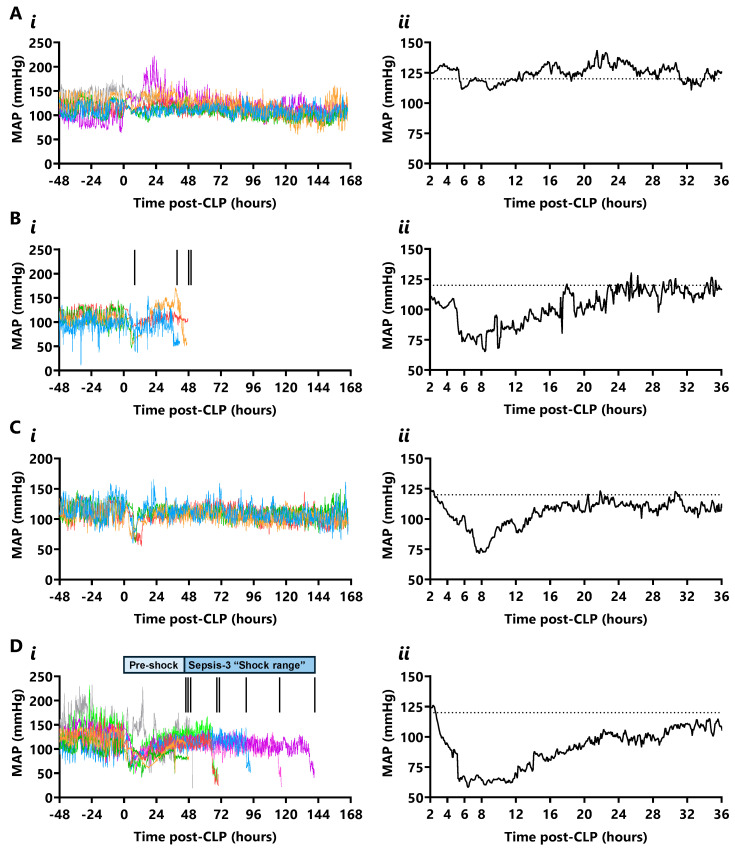
**Real-time mean arterial pressure (MAP) in standard- and high-grade CLP.** (**A**) MAP (mmHg) in standard-grade CLP survivors over (**i**) 168 h (each colour represents a mouse) and (**ii**) mean MAP in the first 36 h after CLP (n = 6). (**B**) MAP in standard-grade CLP non-survivors over (**i**) 168 h and (**ii**) mean MAP over the first 36 h (n = 4; vertical inset lines indicate death time). (**C**) MAP in high-grade CLP survivors (n = 12) over (**i**) 168 h and (**ii**) mean MAP in the first 36 h (n = 4). (**D**) MAP in high-grade CLP non-survivors over (**i**) 168 h and (**ii**) mean MAP in the first 36 h (n = 8). Horizontal dotted line depicts the 120 mmHg median MAP in the 48 h prior to CLP (n = 22).

Real-time mean arterial pressure (MAP) is shown in Figure 2. The ultradian MAP in the 48 h preceding CLP varied between 96 ± 5 mmHg and 143 ± 6 mmHg, with an average period of 88 ± 10 min and 12 cycles per 24 h. This rhythm was lost for 24 h after CLP, then was regained at lower power by 48 h in all mice (Supplementary Figure S1).

Hypotensive episodes, defined by a 40 mmHg MAP drop (33% [three standard deviations] below the 48 h pre-CLP diurnal mean) within 10 min, featured in the period 3.5 to 39 h after CLP. Most standard-grade CLP mice did not become hypotensive (7/10); however, 3/4 non-survivors became hypotensive at 5.5 ± 0.9 h for 6.1 ± 1.2 h with a MAP nadir of 46 ± 9 mmHg (Figure 2(Ai,Aii)). Of these, one died after a single hypotensive episode (beginning at 7.3 h) and two died after two episodes (the second beginning at 36.5 and 44.5 h, Figure 2(Bi,Bii)). Hypotension in these standard-grade mice lasted 3.6 ± 0.3 h before early euthanasia, with a final MAP of 58 ± 3 mmHg. In contrast, two-thirds of high-grade CLP mice (8/12) had two or more hypotensive episodes, which was always lethal, while a third (4/12) survived a single episode. Hypotension began at 6.2 ± 1.6 h (range 4.5 to 10.5 h) in high-grade CLP survivors and lasted 4.8 ± 1.0 h with a MAP nadir of 61 ± 5 mmHg (Figure 2(Ci,Cii)), while, in non-survivors, began at 4.8 ± 1.4 h (range 3.5 to 7.2 h) and lasted 12.3 ± 2.1 h with a MAP nadir of 42 ± 7 mmHg (Figure 2(Di,Dii)). The onset time of hypotension and the MAP nadir did not differ between or within standard- and high-grade CLP cohorts; however, the duration of the first episode was 7.5 h longer in high-grade non-survivors than survivors (*p* ≤ 0.05). Septic shock, defined as a 40 mmHg MAP decrease concurrent with lactate elevation (20% [one standard deviation] above the diurnal mean in unoperated control mice), was not observed in standard-grade CLP mice. However, two-thirds (8/12) of high-grade CLP mice entered septic shock, which was always lethal (non-survivors). Septic shock began at 41.2 ± 0.5 h (three mice), 62.7 ± 0.2 h (two mice), and at 90.2, 110.5, and 137.1 h in single mice. The septic shock-related MAP decrease lasted 7.3 ± 0.5 h until early euthanasia, with a final MAP of 38 ± 8 mmHg.

### 2.3. Heart Rate

**Figure 3:**
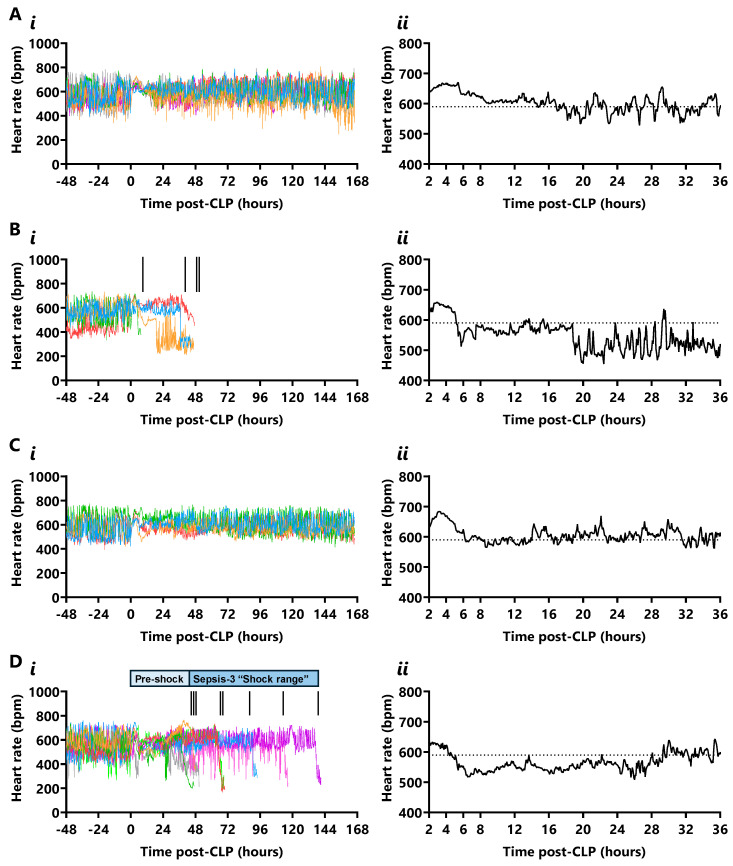
**Real-time heart rate in standard- and high-grade CLP mice**. (**A**) Heart rate (bpm) in standard-grade CLP survivors over (**i**) 168 h (each colour represents a mouse) and (**ii**) mean heart rate in the first 36 h after CLP (n = 6). (**B**) Heart rate in standard-grade CLP non-survivors over (**i**) 168 h and (**ii**) mean heart rate in the first 36 h (n = 4; vertical inset line indicates a death). (**C**) Heart rate in high-grade CLP survivors fitted with telemetry (n = 12/28) over (**i**) 168 h and (**ii**) mean heart rate in the first 36 h (n = 4). (**D**) Heart rate in high-grade CLP non-survivors over (**i**) 168 h and (**ii**) mean heart rate in the first 36 h (n = 8). Horizontal dotted line depicts the median heart rate (590 bpm) in the 48 h prior to CLP (n = 22).

Real-time heart rate is shown in Figure 3. The ultradian heart rate in the 48 h preceding CLP varied between 408 ± 11 bpm and 712 ± 9 bpm, with an average period of 85 ± 13 min and 12 cycles per 24 h. Mice lost this rhythm for 24 h after standard-grade CLP and for 48 h after high-grade CLP, followed by respective normalisation by 48 h and 72 h (Supplementary Figure S2).

A heart rate drop, defined as a 100 bpm decline (17% [three standard deviations] below the pre-CLP diurnal mean) within 10 min, was not observed in any CLP survivor, despite high-grade CLP survivors exhibiting episodes of hypotension (Figure 3A,C). A 140 bpm drop in heart rate (*p* ≤ 0.05) was evident from 7 to 48 h in standard-grade CLP non-survivors, likely due to deterioration leading to early euthanasia at 8 and 48 h (Figure 3(Bi,Bii)). Of the standard-grade CLP non-survivors, 2/4 mice showed a heart rate drop to 326 bpm (*p* ≤ 0.05) at the onset of their final hypotensive episode at 7.3 h (lasting 2.3 h prior to early euthanasia) and 37.7 h (lasting 4.8 h prior to early euthanasia), while 1/4 showed a heart rate drop to 298 bpm (*p* ≤ 0.05) at 18.7 h (lasting 22.6 h prior to early euthanasia), 17.8 h before the onset of their second and final hypotensive episode. In contrast, 1/4 standard-grade non-survivors did not exhibit a heart rate drop before in-cage death. All non-surviving high-grade CLP mice showed a 55% decrease in heart rate to 261 ± 18 bpm (*p* ≤ 0.01) that began 1.7 ± 0.4 h after septic shock-related MAP decline; these mice had a final heart rate of 195 ± 26 bpm (Figure 3(Di,Dii)).

### 2.4. Temperature

**Figure 4:**
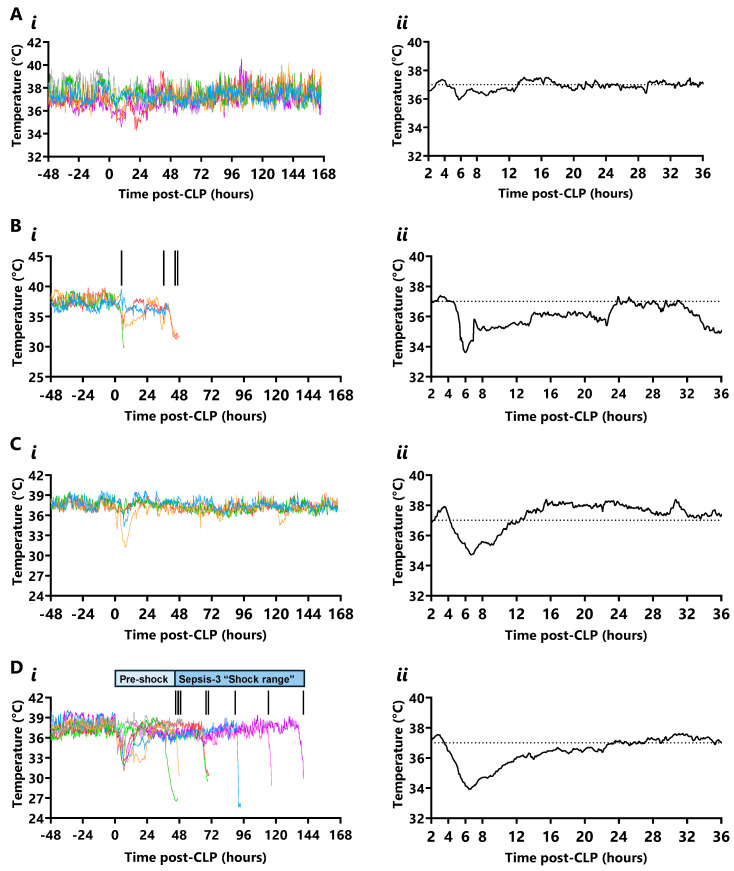
**Real-time core temperature in standard- and high-grade CLP mice**. (**A**) Core temperature (°C) in standard-grade CLP survivors over (**i**) 168 h (each colour represents a mouse) and (**ii**) mean temperature in the first 36 h after CLP (n = 6). (**B**) Core temperature in standard-grade CLP non-survivors over (**i**) 168 h and (**ii**) mean temperature in the first 36 h (n = 4; vertical inset line indicates a death). (**C**) Core temperature in high-grade CLP survivors fitted with telemetry (n = 12/28) over (**i**) 168 h and (**ii**) mean temperature in the first 36 h (n = 4). (**D**) Core temperature in high-grade CLP non-survivors over (**i**) 168 h and (**ii**) mean temperature in the first 36 h (n = 8). Horizontal dotted line depicts the median core temperature (37 °C) in the 48 h prior to CLP (n = 22).

Real-time calibrated core temperatures (Supplementary Table S1) are shown in Figure 4 and ranged between 36.1 ± 0.1 °C and 38.0 ± 0.2 °C with an average period of 22 ± 2 h and a single cycle per 24 h in the 48 h preceding CLP; this rhythm was lost for 24 h following CLP and regained by 48 h in all mice (Supplementary Figure S3).

Calibrated core temperatures after CLP were defined as mild hyperthermia above 38.0 °C, mild hypothermia between 35.5 and 32.0 °C, and hypothermia below 32.0 °C. Standard-grade CLP survivors displayed heterogeneous temperature profiles; half maintained temperatures within the normal diurnal range, while half developed mild hypothermia with a nadir of 35.1 ± 0.7 °C at 6.0 ± 0.8 h after CLP, lasting 6.1 ± 0.8 h (Figure 4(Ai,Aii)). Mild hypothermia was also evident in standard-grade CLP non-survivors at 5.6 ± 1.1 h to a nadir of 33.5 ± 1.4 °C and a duration of 5.7 ± 1.5 h, which occurred 1.8 ± 1.3 h prior to the onset of the first hypotensive episode (Figure 4(Bi,Bii)). In contrast, an initial mild hyperthermia before an extended period of hypothermia, then recovery, was noted in all high-grade CLP mice. Mild hyperthermia was evident in high-grade CLP survivors to a peak of 38.1 ± 0.2 °C at 4.1 ± 0.3 h before a transition to hypothermia at 5.2 ± 0.3 h (onset range 3.1 to 6 h; 1.1 ± 0.1 h before the first hypotensive episode) with a nadir of 34.5 ± 1.2 °C at 6.0 ± 0.9 h; temperature then normalised at 11.2 ± 0.9 h (Figure 4(Ci,Cii)). A similar profile was apparent in high-grade CLP non-survivors, with a temperature rise to 37.8 ± 0.2 °C at 3.8 ± 0.4 h, then transition to hypothermia at 4.3 ± 0.2 h (onset range 3.0 to 6.1 h; 0.5 ± 0.1 h before the first hypotensive episode) with a nadir of 34.0 ± 0.7 °C at 6.7 ± 0.5 h; temperature then normalised at 12.4 ± 0.7 h (Figure 4(Di,Dii)).

Despite avoiding septic shock, temperature declined to 35.2 ± 0.1 °C in 3/10 non-surviving standard-grade CLP mice 4.7 ± 0.5 h before the onset of the second, final hypotensive episode (lasting 8.3 ± 0.4 h prior to early euthanasia) with a final measured temperature of 30 ± 0.1 °C. In contrast, temperature declined to 34.9 ± 0.1 °C at 1.6 ± 0.4 h prior to the septic shock-related MAP drop in all high-grade non-survivors, with a final temperature of 28.3 ± 0.6 °C at early euthanasia.

### 2.5. Time Course Series

#### Plasma Damage and Stress Markers

**Figure 5:**
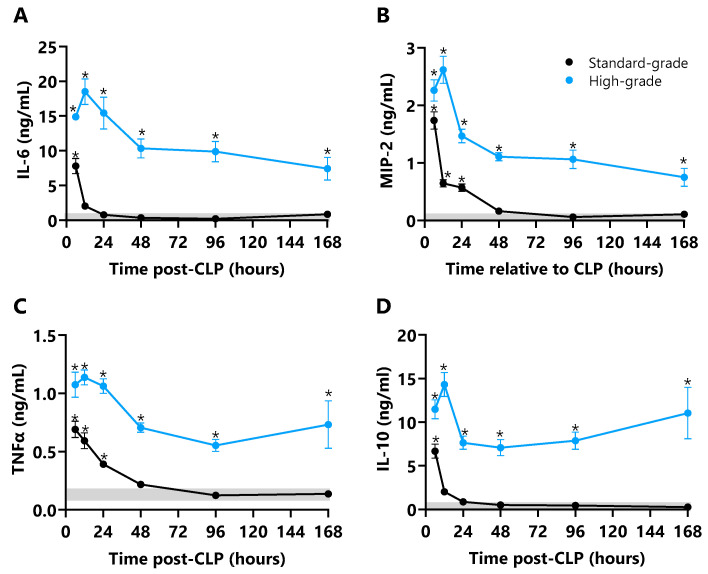
**Plasma cytokine responses in standard- and high-grade CLP mice**. Plasma (**A**) interleukin (IL)-6 (ng/mL), (**B**) IL-10 (ng/mL), (**C**) macrophage inflammatory protein-2 (MIP-2; ng/mL), and (**D**) tumour necrosis factor alpha (TNFα; pg/mL) concentrations at 6, 12, 24, 48, 96, and 168 h after standard- and high-grade CLP (n = 6 per time point). Horizontal grey bar depicts the diurnal range of each cytokine measured in unoperated control mice (n = 24). Data are presented as mean ± SEM; * *p* ≤ 0.05.

Plasma lactate, albumin, and total corticosterone profiles in CLP mice are shown in Figure 5. Lactate concentrations did not change in standard-grade CLP mice but increased in high-grade CLP mice from 48 h (39%) to 168 h (71%, all *p* ≤ 0.001 vs. unoperated control mice, Figure 5A). This increase, in conjunction with MAP decline, marked the onset and progression of septic shock. Non-surviving mice (early euthanasia) had higher lactate concentrations than survivors only after high-grade CLP (21%, *p* ≤ 0.05).

Albumin concentrations decreased in all CLP mice relative to unoperated control mice, spanning a 48 h nadir (41%, *p* ≤ 0.01) to 96 h (32%, *p* ≤ 0.05) before a return to normal in standard-grade CLP mice at 168 h. Plasma albumin declined from 24 h (37% *p* ≤ 0.05) to a 48 h nadir (55%, *p* ≤ 0.001) that coincided with septic shock onset in high-grade CLP mice, with no recovery to 168 h (49%, *p* ≤ 0.001, Figure 5B). Non-surviving mice (early euthanasia) had lower plasma albumin than survivors after standard- (70%, *p* ≤ 0.001) and high-grade CLP (59%, *p* ≤ 0.001, Table 1).

Plasma total corticosterone was unchanged after standard-grade CLP yet increased relative to unoperated control mice in high-grade CLP mice from 6 h (97%, *p* ≤ 0.001) to a 12 h peak (165%, *p* ≤ 0.001), then declined to 48 h and plateaued to 168 h coincident with septic shock onset and progression (71%, *p* ≤ 0.001, Figure 5C). Non-surviving mice (early euthanasia) had higher total corticosterone than survivors after both standard- (194%, *p* ≤ 0.05) and high-grade CLP (106%, *p* ≤ 0.001).

### 2.6. Plasma Cytokines

**Figure 6:**
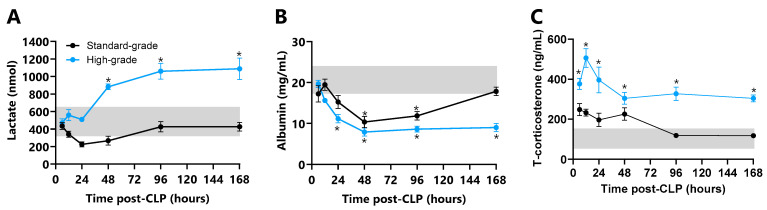
**Plasma lactate, albumin, and total corticosterone in standard- and high-grade CLP mice**. Plasma (**A**) lactate (nmol), (**B**) albumin (mg/mL), and (**C**) total corticosterone (ng/mL) concentrations at 6, 12, 24, 48, 96, and 168 h after standard- and high-grade CLP (n = 6 per time point). Horizontal grey bar depicts the diurnal range of individual plasma markers measured in unoperated control mice (n = 24). Data are presented as mean ± SEM; * *p* ≤ 0.05.

Plasma IL-6, MIP-2, TNFα, and IL-10 profiles in CLP mice are shown in Figure 6. IL-6, MIP-2, TNFα, and IL-10 concentrations peaked at 6 h in standard-grade CLP mice at levels 37-, 39-, 17-, and 7-fold higher than unoperated control mice (all *p* ≤ 0.001); IL-6 and IL-10 then normalised to control levels by 12 h, while MIP-2 and TNFα normalised by 48 h (Figure 6). IL-6, MIP-2, TNFα, and IL-10 concentrations were markedly elevated by 6 h in high-grade CLP mice, and peaked at 12 h with levels 105-, 30-, 9-, and 89-fold higher than control mice (all *p* ≤ 0.001) aligned to the onset of the first hypotensive episode. Cytokine concentrations then declined to 48 h in high-grade CLP mice, then maintained a plateau phase to 168 h for IL-6 (56-fold, *p* ≤ 0.001), MIP-2 (12-fold, *p* ≤ 0.001), TNFα (4-fold, *p* ≤ 0.01), and IL-10 (8-fold, *p* ≤ 0.001; Figure 6). The period of septic shock onset and progression was marked by progressive decline in IL-6 and MIP-2, and progressive increase in TNF-α and IL-10. CLP non-survivors had markedly elevated plasma IL-6 levels compared to survivors after standard- (5.2-fold, *p* ≤ 0.001) and high-grade CLP (56%, *p* ≤ 0.001); non-surviving mice also had a marked increase in TNFα after high-grade CLP (44%, *p* ≤ 0.05, Table 1).

### 2.7. Plasma Markers of Organ Damage

**Figure 7:**
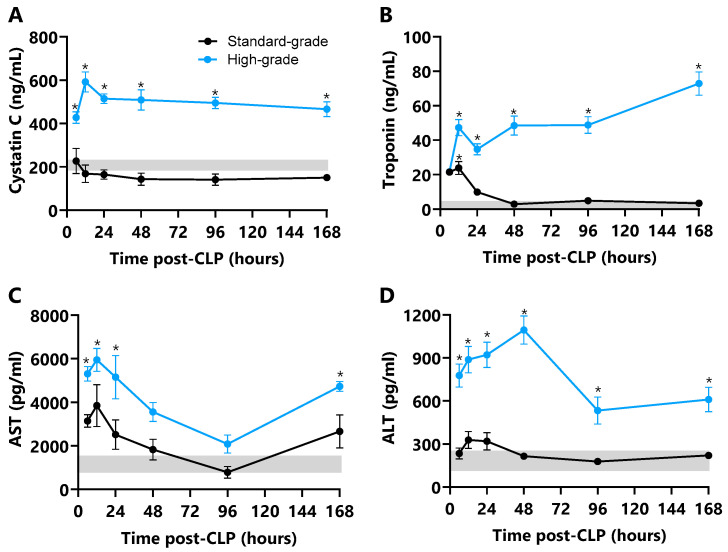
**Plasma markers of organ damage in standard- and high-grade CLP mice**. Plasma (**A**) cystatin C (ng/mL), (**B**) troponin I (ng/mL), (**C**) aspartate transaminase (AST; pg/mL), and (**D**) alanine transaminase (ALT; pg/mL) concentrations at 6, 12, 24, 48, 96, and 168 h after standard- and high-grade CLP (n = 6 per time point). Horizontal grey bar depicts the diurnal range of individual damage marker measured in unoperated control mice (n = 24). Data are presented as mean ± SEM; * *p* ≤ 0.05.

Plasma cystatin C, AST, and ALT concentrations did not change in standard-grade CLP mice relative to unoperated control mice (Figure 7A,C,D). Troponin-I, however, increased to a 12 h peak (6-fold, *p* ≤ 0.001) then normalised by 24 h (Figure 7B). High-grade CLP markedly increased plasma cystatin-C and AST, with both peaking 3-fold at 12 h (*p* ≤ 0.001; Figure 7A,B), coinciding with the first hypotensive episode. Cystatin-C remained elevated at 168 h (2-fold, *p* ≤ 0.001), whereas AST normalised between 48 and 96 h before a rise to a 3-fold increase at 168 h (*p* ≤ 0.001). Troponin-I increased, then stabilised from 12 h to 96 h (14-fold, *p* ≤ 0.001) before a further increase to peak at 168 h (22-fold, *p* ≤ 0.001, Figure 7B); ALT increased from 12 h to a peak at 48 h (4-fold, *p* ≤ 0.001) before a decrease to lower but elevated concentrations at 96 h and 168 h (2-fold, *p* ≤ 0.001, Figure 7D). The period of septic shock onset and progression was marked by stable cystatin-C, a progressive increase in troponin-I, and dynamic AST and ALT. These plasma markers did not differ between standard- or high-grade CLP survivors and non-survivors (Table 1).

### 2.8. Summary Results

**Figure 8:**
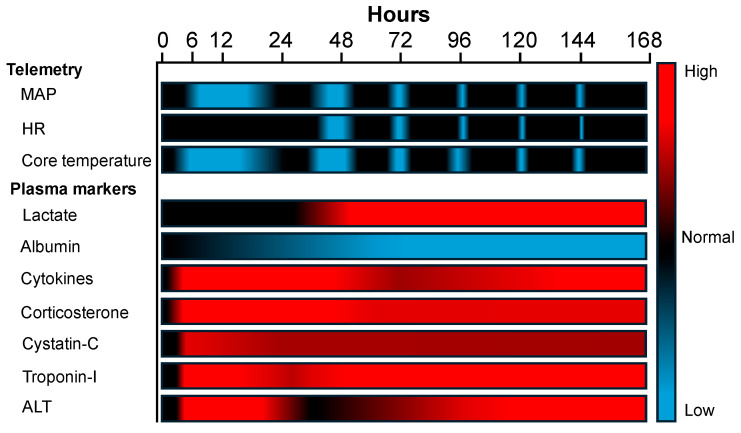
Heat map summary of telemetry and plasma biomarker progression following CLP. Blue indicates low reading (telemetry) or plasma marker levels; red indicates low reading (telemetry) or plasma marker levels; black indicates normal physiological range. MAP, mean arterial pressure; HR, heart rate; ALT, alanine aminotransferase. Note: The blue shades for telemetry data from 40 h onwards denote progression to septic shock.

A graphical heat map summary of the telemetry and plasma biomarker results is illustrated in Figure 8.

## 3. Discussion

We have characterised murine models of polymicrobial sepsis and septic shock following standard- and high-grade CLP to precisely map the onset of hypotension and septic shock and its progression using arterial telemetry and a repertoire of plasma biomarkers. Our standard- and high-grade CLP mice modelled the range of mortality expected in the absence of ventilatory and/or pressor support provided in an ICU [5]. All high-grade CLP mice developed MAP-defined hypotension and most progressed to septic shock. In line with the Sepsis-3 consensus [4], we defined murine septic shock as the coincidence of persistent hypotension and elevated plasma lactate. This definition was applied despite fluid resuscitation, a key component of clinically relevant septic shock management, which precluded normovolemia as a defining criterion. CLP mice showed stark grade-dependent differences; standard-grade CLP led to transient or modest changes in plasma cytokines and markers of systemic and organ damage, while high-grade CLP led to marked and sustained increases (and a decrease in albumin), indicating progressive multiple organ damage. Our refined CLP models are critical as they recapitulate the clinical heterogeneity of human sepsis, offering a spectrum of injury severity ranging from non-lethal to overwhelming lethal septic shock. Alternative models, such as faecal slurry peritonitis, can be suboptimal due to limitations in severity control or a lack of acute onset [25]. Conversely, other promising models, such as colon ascendens stent peritonitis, entail higher surgical demand and are consequently yet to be widely adopted [26]. Nevertheless, CLP remains broadly recognised as the gold standard [14] due to its established methodology and extensive historical data, while our refined CLP models specifically enable real-time mapping of acute physiological deterioration.

These refined caecal ligation and puncture (CLP) models are critical as they offer a spectrum of injury severity, reliably mirroring the clinical heterogeneity of human sepsis, from non-lethal to overwhelming lethal septic shock. In contrast to models of low-grade sepsis (e.g., colon ascendens stent peritonitis) or non-surgical insult (e.g., faecal slurry peritonitis), which are often inferior due to limited severity control or a lack of acute onset, our CLP models allow for the real-time mapping of acute physiological deterioration using arterial telemetry and a repertoire of plasma biomarkers, enabling precise study of the septic shock trajectory.

### 3.1. Survival, Morbidity, and Weight

Mortality, morbidity, and weight loss were higher after high-grade CLP than standard-grade CLP in this study, as expected. CLP outcomes are strongly influenced by caecal ligation length, the number of punctures [18], mouse strain [27], sex [28] and age [29], and post-operative care paradigm [30]. Indeed, low morbidity and mortality of 20–50% at 4–10 days [18,29,31], and minor weight loss of 5–6% with 72 to 96 h recovery are reported after standard-grade CLP of 8 to 15 mm caecal ligation and 21G puncture [20,32,33,34]. In contrast, reports in standard to high-grade CLP models with greater than 15 mm ligation and 20G puncture show increased morbidity, a mortality of 60–100% within 4–7 days, and sustained weight loss of 10 to 15% in survivors [35,36], on a background of varied postoperative care [18,29,31]. These reported values are within the range reported for standard and high-grade CLP in this study.

### 3.2. Mapping CLP Sequelae with Wireless Telemetry

To our knowledge, the application of wireless telemetry in murine CLP is limited to one study, where device implantation and CLP (10 mm ligation, 21G dual punctures) were performed concurrently [37]. This contrasts with the 4-day recovery period implemented in our protocol to isolate the physiological sequelae of CLP. Notably, the previous study, which did not report real-time MAP, used a sustained 10% drop from peak heart rate and the differential between peak core temperature and 25 °C to define a threshold onset of acute physiologic deterioration, which spanned 16.5 h (5.9 to 22.4 h) after CLP [37]. This study, however, did not specify the qualifying duration of the heart rate drop and used a circular validation of this threshold. In the current study, we observed mild, transient hypertension for 24 h following telemetry implantation. This effect, indicative of acute surgical stress, resolved completely by 48 h (two days prior to the CLP procedure). Furthermore, our pilot studies demonstrated no increase in inflammatory markers at 48 h after telemetry implantation, indicating that baseline inflammation had subsided. This evidence supports the scheduling telemetry implantation sufficiently early effectively eliminates acute physiological confounding effects prior to the CLP procedure.

In this study, hypotension was defined by a MAP fall of 40 mmHg within 10 min and began around 7 h (3.5 to 10.5 h) after high-grade CLP. This first hypotensive episode was preceded by a core temperature decline of around 1 h in high-grade CLP mice, which reached the hypothermia criterion (below 35.5 °C) synchronous with the onset of the MAP decline. These findings support use of the initial temperature decline as a predictor for subsequent hypotension. The synchronous occurrence of these two events at the hypothermia threshold serves as a precision indicator of the acute physiological deterioration following high-grade CLP. In contrast, real-time heart rate was an unreliable marker for acute physiological deterioration, or of septic shock; a third of high-grade CLP mice survived a hypotensive episode without a change in heart rate, while heart rate only fell below the diurnal control range around 2 h after the onset of septic shock in high-grade CLP non-survivors, prior to early euthanasia.

Our use of telemetry has defined these early features as previously unmapped antecedents of sepsis, coincident with rapid hyperdynamic cytokine responses, corticosterone rise, organ damage onset, and early morbidity, likely caused by acute vasodilation, myocardial depression, microvascular injury, and tissue hypoperfusion. It should be noted that despite similar timing of hypothermia onset to other reports of standard and high-grade murine CLP at 6–8 h [38,39], the core temperature decline in this study was variable and correlated with survival after standard-grade CLP; as such, it is likely less predictive of acute physiological deterioration in lower impact CLP models.

The temperature drop in CLP mice is likely due to failed thermoregulation, driven by an acute inflammatory response marked by elevated IL-6 and TNFα. Indeed, a primary role of the TNF axis in the onset of sepsis hypothermia is well supported; the TNF receptor 1 (TNFR1) and matrix metalloproteinase 8 (MMP8, which cleaves pro-TNFα into its bioactive form) are required for typical hypothermia following acute lipopolysaccharide (LPS) endotoxemia or polymicrobial CLP in mice, whereas mice deficient in both TNFR1 and MMP8 did not exhibit hypothermia and were fully protected [40,41]. The rapid rise in plasma TNFα here through 6 to 24 h coincided with hypothermia onset in high-grade CLP mice, which resolved after 24 h in survivors when TNFα began to fall. Indeed, all high-grade CLP non-survivors had profound hypothermia below 30 °C and significantly higher TNFα than survivors. However, it should be noted that, in contrast to hypothermia in CLP mice, patients with sepsis and septic shock present with hypothermia or hyperthermia, with the former linked to a poorer prognosis [42].

While the coincidence of hyperthermia and hypotension was an early CLP sequala prior to 48 h in high-grade CLP mice, its coincidence with lactate elevation and severe hypotension (below 60 mmHg) from 48 h defined the onset of universally lethal septic shock (and the peak window for early humane euthanasia). Core temperature began to decline around 1.5 h before this hypotensive episode and met the criterion of hypothermia (below 35.5 °C) synchronous with the onset of the MAP fall.

The primary pathological mechanism in septic shock is systemic vasodilation. While cardiac output can initially compensate, it often fails later in the progression of septic shock, evidenced here by a heart rate decline that lagged behind the MAP drop by around 2 h. This finding aligns with clinical practice, where a drop in MAP to below 65 mmHg [43], or a fall of 40 mmHg from baseline [44] (if measured), necessitates immediate vasopressor support [45,46]. Without this support, septic shock is often lethal [47].

In summary, our use of wireless telemetry here has permitted an unprecedented temporal view of physiological deterioration from murine CLP, spanning newly mapped early hypotensive episodes to septic shock, and the heterogeneous range that sepsis presents clinically.

### 3.3. Cytokines

Nominally pro- (IL-6, MIP-2, TNFα) and anti-inflammatory cytokines (IL-10) form a double-edged sword in sepsis and septic shock [48] and are required to respond to, and eliminate infection, while excessive production causes organ damage [49]. This cytokine storm was transient in standard-grade CLP, peaking at 6 h and normalised by 48 h, consistent with rapid normalisation of plasma markers of cardiac and liver damage. However, the cytokine peak in high-grade CLP mice was seen later, at 12 h, and remained elevated to demark the model, injury, and mortality difference. It is well described that caecal ligation length dictates the cytokine response in CLP mice, with full-length ligation (20–30 mm) in C57BL/6 mice known to result in an exaggerated cytokine response led by IL-6 and TNFα compared to a 5–7 mm ligation [18]. Species differences in cytokine response to CLP are also known, with high IL-6 concentrations linked to mortality in Harlan-Sprague Dawley mice [50], while high TNFα is seen in C57BL/6 and CD-1 mice non-survivors [51,52]. Consistent with this, high-grade CLP non-survivors in this study had higher plasma IL-6 and TNFα than survivors. However, in contrast to evidence of high plasma MIP2 in CD-1 mice non-survivors after full-length CLP [53], there was no difference in MIP-2 response or concentration between high-grade CLP survivors and non-survivors in this study, likely related to species or CLP procedure differences [18,54].

A similar CLP response profile for all plasma cytokines was seen over 6 to 96 h, with a distinct and late increase to 168 h for TNFα and IL-10 that coincided with hyperlactatemia and worsening cardiac and liver damage. IL-6 and TNFα both trigger the production and release of IL-10 [55,56] and TNFα may well drive the late phase IL-10 response in mice together with sustained IL-6 production. Plasma IL-10 also increases in patients with septic shock and is positively associated with inflammation severity and the development of multi-organ failure [57], likely due to IL-10 following pro-inflammatory cytokine responses.

### 3.4. Systemic Damage Markers

Hyperlactatemia (an indicator of tissue hypoperfusion) and hypoalbuminemia (an indicator of infection, liver damage, or kidney damage) are clinically used to index sepsis severity and serve as independent markers of mortality risk [36,58]. Lactate rose only in high-grade CLP mice, and after the acute hyperdynamic phase marked by hypotensive episodes and peak cytokine. This concurrent rise in lactate and MAP drop marked the onset of septic shock, with higher lactate evident in CLP non-survivors than survivors, consistent with findings in Sprague-Dawley rats [36]. The lactate rise after CLP was delayed relative to a report in C57BL/6 mice, where a 6 h hyperlactatemia followed a full-length CLP [18], underscoring the importance of injury severity in this model. This may also relate to differences in fluid resuscitation, which prevent or delay hyperlactatemia in patients [59,60] and rats [61], and were postoperative compared to ongoing in this study. Of note, the sustained lactate increase coincided with cardiac troponin-I in high-grade CLP mice, a relationship known to be associated with significant myocardial injury clinically [62,63].

Albumin, in contrast, was lower in all CLP mice and in non-survivors compared to survivors and remained attenuated in high-grade CLP mice. Until this study, knowledge of albumin responses after murine CLP had been limited to a report of no change at 24 h after standard-grade CLP [64], as here. Importantly, the sustained decline in albumin after high-grade CLP accords with clinical evidence, where such a decrease is associated with increased sepsis severity and mortality [58,65].

### 3.5. Corticosterone Response

As mice lack the adrenal 17α-hydroxylase enzyme required to synthesise cortisol, corticosterone serves as the main glucocorticoid within the HPA axis, and effectively modulates hepatic gluconeogenesis, stress responses, and anti-inflammatory immune functions [66,67,68]. Total corticosterone in standard-grade CLP mice did not change relative to the diurnal range mapped in unoperated control mice, but rapidly increased in high-grade CLP mice and remained elevated, reflecting injury grade differences; high levels were also apparent in CLP non-survivors compared to survivors. This adds support that the corticosterone elevation in high-grade survivors represents an adaptive HPA axis response, intended to provide requisite immune support. Conversely, the higher corticosterone levels observed in non-survivors likely represent pathological dysregulation or a failure of the axis to manage the overwhelming injury and inflammation. A rapid increase in plasma total corticosterone has been reported within 2 h of CLP in C57BL/6 mice [69] or after LPS administration in Balb/C mice [70] in the few studies to have assessed this measure. Increased total cortisol in patients with sepsis is linked to increased mortality risk [71]; however, elevated free cortisol may best predict the severity and mortality risk in patients with sepsis [72,73], and was not measured as a study limit here due to the high plasma volume requirement of the spin column analysis.

### 3.6. Organ Damage Markers

Multi-organ failure is a hallmark of severe sepsis and occurs secondary to inflammation-induced tissue hypoxia and cell damage [74]. Cardio-circulatory and microcirculatory dysfunction are the main drivers of tissue hypoperfusion in septic shock and key to the development of multi-organ failure [75,76]. Cystatin C, troponin-I, and ALT were significantly elevated at 6 h after high-grade CLP in this study, as well as troponin-I in standard-grade CLP, reflecting the onset of kidney, cardiac, and liver damage, respectively; these elevations were transient in standard-grade CLP, yet persisted in high-grade CLP mice and likely coincide with robust progression to multi-organ failure. Unexpectedly, we found that organ damage markers, per se, poorly predicted survivorship within standard- and high-grade CLP mice cohorts. This adds focus to the higher peak and persistence of pro-inflammatory cytokines IL-6 and TNFα, hyperlactatemia, hypoalbuminemia, and corticosterone, as key mechanisms underlying non-survivorship after high-grade CLP.

Past use of sham mice as CLP controls has documented a mild, transient inflammation with mild cytokine increases [77,78]. We chose to precisely map the diurnal range of all biomarkers in healthy, un-operated mice for control baseline, allowing distinction to modest inflammatory features after low-grade CLP and to severe and sustained biomarker elevations to 1000-fold beyond those in sham or low-grade CLP after high-grade CLP, as a clinically relevant model of severe human sepsis.

## 4. Materials and Methods

### 4.1. Ethics

This study was approved by the Animal Ethics Committee of the South Australian Health and Medical Research Institute (SAHMRI, as SAM-21-024) and carried out in compliance with the Australian Code for the Care and Use of Animals for Scientific Purposes (8th Edition) and the ARRIVE guidelines [79].

### 4.2. Animals

Adult (10–12 week old; 25–30 g) male C57BL/6 mice (n = 122, bred in SAHMRI Bioresources) were housed individually in a 12:12 h light–dark cycle under constant temperature (22 ± 0.5 °C) and humidity (40–60%) and had ad libitum access to water and a standard chow diet (13 kJ/g: 24% from protein, 18% from fat, 58% from carbohydrates; #2918, Teklad Global Diet, Indianapolis, IN, USA). Male mice were used in this study to eliminate confounding effects from the oestrus cycle on all measurements; future studies will map disease progression and outcomes in relation to the oestrus cycle stage.

### 4.3. Surgical Procedures

#### 4.3.1. Carotid Telemetry

Mice were fasted for 2 h (0630-0830), then anesthetised with 2.5% isoflurane in oxygen (0.5 L/min) and placed in the supine position on a heating pad. A 1 cm incision was made 1–2 mm lateral to the trachea, and the right common carotid artery was located and isolated from surrounding tissues. A 6-0 silk ligature (#18020-60; Fine Science Tools, Foster City, CA, USA) was then tied inferior to the cephalic bifurcation, and a microvascular clamp (#RS-5440; Roboz, Gaithersburg, MD, USA) was placed 5–8 mm below to temporarily block carotid flow. A small transverse incision (microdissection scissors #RS-5618; Roboz, Gaithersburg, MD, USA) was made in the artery 2 mm below the bifurcation and the catheter of the HD-X10 wireless telemetry device (#270-0171-002X; Data Science International, New Brighton, MN, USA) was inserted into the carotid artery, secured with a loose 6-0 silk ligature, then advanced beyond the microvascular clamp (upon clamp removal) to a position 1–2 mm into the aortic arch, marked by a notch on the catheter (position standardised in age and weight-matched mice). The catheter was then secured with dual 6-0 silk ligatures and the telemetry body tunnelled under the skin to the right abdominal flank. The neck incision was closed with interrupted 6-0 non-absorbable polypropylene monofilament sutures (#PM196016F11M; eSutures, Will County, IL, USA) then 1 mg/kg of buprenorphine and fluid (1 mL warmed saline) were administered subcutaneously before mice were removed from anaesthesia, returned to individually ventilated cages (IVCs) with wetted food pellets, enrichment and in-cage warming (24 h heat pad under cage). A 4-day recovery period preceded further intervention, during which daily weight and blood pressure were monitored.

#### 4.3.2. Caecal Ligation and Puncture (CLP)

**Figure 9:**
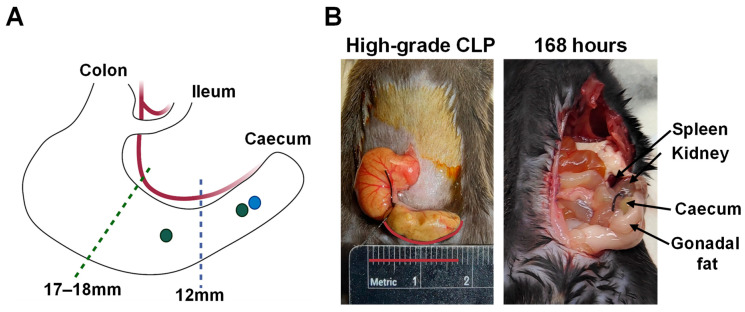
**Caecal ligation and puncture (CLP) procedure**. (**A**) Mouse caecum depicting ligation lengths and puncture/s related to standard (blue) and high-grade (green) CLP. (**B**) In-operative and 168 h post-operative image of high-grade CLP.

Mice were randomised to standard- or high-grade CLP groups via a computer-generated block random number sequence. Mice were then fasted for 2 h (0630–0830), and then anaesthetised with 2.5% isoflurane in oxygen (0.5 L/min). A 1–2 cm midline abdominal laparotomy was performed, and the caecum was located and exteriorised using saline-wetted cotton swabs. Caecal content was distributed evenly, air removed, then the caecum was ligated with a 4-0 silk ligature (#A303H, Ethicon, North Ryde, Australia) and punctured with a 21G needle to model standard-grade (11–12 mm ligated caecum length, single pass puncture) or high-grade CLP (17–18 mm ligated caecum length, two single pass punctures; see Figure 9). A 1 mm column of faeces was then extruded from each puncture site to ensure patency. The caecum was then returned to the peritoneal cavity, the muscle layer closed with interrupted 6-0 non-absorbable prolene sutures (eSutures), and the skin closed using 7 mm wound clips (RS-9258, Roboz, Gaithersburg, MD, USA). Mice were administered postoperative buprenorphine (0.1 mg/kg; #TemVet, Troylab, Glendenning, Australia), enrofloxacin (1 mg/100g; #1008343, ChemPro, Ashmore, Australia), and warmed saline (1 mL), via subcutaneous injection, then returned to individual home IVCs with wetted food pellets, enrichment, and under-cage warming (12 h).

### 4.4. Post-Operative Care Regimen

Mice were monitored every 8 h (0700, 1500, 2300) to 168 h and scored for body condition, real-time hypothermia (below 30 °C) or hyperthermia (above 38 °C), weight loss, reduced mobility, diarrhoea, abdominal distension, laboured breathing, or loss of righting reflex for a validated cumulative disease index (CDI) [19]; laboured breathing or loss of righting reflex was set as threshold for euthanasia. All mice were administered subcutaneous buprenorphine (0.1 mg/kg) every 8 h for 48 h and warmed saline (1 mL) every 12 h for 168 h, with subcutaneous enrofloxacin (1 mg/100 g) administered every 8 h to 168 h to standard-grade CLP mice only.

### 4.5. Survival Series

Survival was mapped over 7 days in standard- (n = 10) and high-grade CLP mice cohorts (n = 28) to determine telemetry shock characteristics (hypotension), morbidity, and mortality. CLP mice surviving at 7 days were fasted for 2 h in wire-bottomed cages (0700-0900), anesthetised (5% isoflurane in 1% oxygen), then euthanised (cardiac exsanguination); mice that met prior threshold criteria were euthanised without fasting. Terminal bleeds were collected in EDTA tubes (1 mL, #450531, Interpath, Melbourne, Australia) and centrifuged at 4 °C and 12,000× *g* for 15 min, with plasma stored at −80 °C.

### 4.6. Time Course Series

A longitudinal assessment of sepsis and septic shock progression was conducted in standard- (n = 30) and high-grade CLP mice (n = 30). Mice were randomised to timed euthanasia and blood collection at 6, 12, 24, 48, or 96 h post-CLP (n = 6 per time point). All mice were fasted for 2 h prior to these timed endpoints and euthanised with blood collection and storage as described above.

### 4.7. Plasma Bioassays

Plasma concentrations of total corticosterone (#80556, Crystal Chem, Elk Grove Village, IL, USA, RRID:AB 3677403), albumin (#80630, Crystal Chem, RRID:AB-3677402), tissue hypoxia marker lactate, (#AB65331, Abcam plc, Cambridge, UK), and organ damage markers of cardiac troponin-I (#LS-F4165, LS Bio, Newark, CA, USA, RRID:AB 3677397), renal cystatin-C, (#AB119590, Abcam, RRID:AB 3677396), liver alanine transaminase (ALT; #AB282882, Abcam, RRID:AB 3094649), and aspartate aminotransferase (AST; #AB263882, Abcam, RRID:AB 3094650) were determined using commercial ELISA kits. Plasma cytokines were determined via a customised MILLIPLEX^®^ Mouse High Sensitivity T Cell Magnetic Bead Panel for TNFα, IL-6, IL-10, and macrophage inflammatory protein-2 (MIP-2, #MHSTCMAG-70K, Merck KGaA, Darmstadt, Germany, RRID:AB 3677398). Assays were conducted according to the manufacturer’s specified protocol. To prevent bias, investigators were blinded to the treatment group assignment and temporal origin of each sample during the experiment and data analysis.

### 4.8. Biological Rhythms

Ultradian rhythms of real-time MAP and heart rate, and circadian rhythms of calibrated core temperature (Supplementary Table S1), were assessed in the 48 h prior to CLP using Cosinor software [80]. To establish a normal range for plasma assay targets, diurnal ranges were measured in separate, individually housed, unoperated control mice that were humanely euthanised for blood collection at CLP-equivalent timepoints of 6, 12, and 24 h (0900, 1500, 2100, and 0900, n = 24).

### 4.9. Statistical Analysis

Survivability was determined using a Kaplan–Meier 7-day survival curve. Weight change, CDI score, and biomarker time course data were assessed using a two-way ANOVA, while post hoc comparisons, with adjusted *p* values for multiple comparisons, were performed with Tukey’s correction to test significant interactions. Plasma biomarker differences between mice survivors and mice that met prior euthanasia threshold were assessed via a time-corrected multivariate analysis of variance with tests of between-subjects effects. A *p*-value equal to or less than 0.05 was considered significant. All data were first tested for normality using the Shapiro–Wilk test and are presented as mean ± standard error of mean (SEM). Analyses were conducted using GraphPad Prism software (v10.0, Dotmatics, Boston, MA, USA) or SPSS software (v29.0, IBM SPSS Inc., Armonk, NY, USA).

## 5. Conclusions

We have refined murine models of sepsis following standard- and high-grade CLP and integrated real-time arterial telemetry with precision plasma biomarkers to monitor circulatory, temperature, and multi-organ changes throughout the progression from sepsis to septic shock. High-grade CLP reliably induced septic shock, characterised by the coincidence of hypothermia, deep hypotension, elevated lactate, marked systemic inflammation, organ damage, and high mortality. These findings validate the robustness of this high-grade model, highlighting its suitability for testing novel therapies to address the need for more effective sepsis and septic shock treatments.

The integration of wireless telemetry enabled real-time tracking of deterioration in CLP mice, providing unprecedented insight into the dynamic course of sepsis, from the previously unmapped coincidence of hypothermia and early hypotensive episodes marking the onset of acute physiological deterioration, to the later onset of septic shock. We advocate for increased adoption of wireless telemetry in sepsis studies to significantly enhance the detection of critical inflection points and the impact of experimental interventions.

## Figures and Tables

**Table 1:** Plasma biomarkers in CLP survivors and non-survivors.

Biomarker	Standard-Grade CLP			High-Grade CLP		
	Survivors	Non-Survivors	*p*-Value	Survivors	Non-Survivors	*p*-Value
**IL-6 (ng/mL)**	0.9 ± 0.1	5.6 ± 2.2	**0.001**	8.9 ± 0.8	13.9 ± 1.4	**0.001**
**MIP-2 (ng/mL)**	0.1 ± 0.02	0.3 ± 0.2	0.532	0.9 ± 0.04	1.2 ± 0.07	0.219
**TNFα (ng/mL)**	0.7 ± 0.2	0.7 ± 0.6	0.992	0.5 ± 0.05	0.9 ± 0.06	**0.022**
**IL-10 (ng/mL)**	0.3 ± 0.04	0.5 ± 0.1	0.617	11.1 ± 2.95	8.8 ± 1.7	0.251
**Lactate (nmol)**	426 ± 49	317 ± 67	0.203	1089 ± 123	1381 ± 73	**0.021**
**Albumin (mg/mL)**	17.8 ± 1.1	5.3 ± 0.1	**<0.001**	9.1 ± 1.2	3.7 ± 0.9	**0.002**
**Corticosterone (ng/mL)**	118 ± 8.0	346 ± 26	**0.023**	304 ± 17	627 ± 38	**<0.001**
**Cystatin C**	150 ± 6	122 ± 18	0.293	466 ± 34	460 ± 27	0.554
**Troponin-I**	3.3 ± 0.4	4.9 ± 0.9	0.057	72.9 ± 6.7	57.6 ± 9.5	0.335
**ALT (pg/mL)**	220 ± 17	209 ± 43	0.562	610 ± 86	625 ± 138	0.916
**AST (pg/mL)**	2666 ± 762	2377 ± 814	0.897	4734 ± 231	3652 ± 637	0.842

IL-6, Interleukin-6; MIP-2, macrophage inflammatory protein-2; IL-10, Interleukin 10; ALT, alanine aminotransaminase; AST, aspartate aminotransferase; Data were analysed correcting for time of death or survival and are presented as mean ± SEM; bold *p* values represent a significant difference.

## Data Availability

Any additional information required to reanalyse the data reported in this paper is available from the lead contact upon request.

## Supplementary Materials

The following supporting information can be downloaded at: https://www.mdpi.com/article/10.3390/ijms26209954/s1.
