# Supplementary material for: Precision Profiling of Disease Progression in Murine Models of Sepsis and Septic Shock
Source: Int J Mol Sci. 2025 Oct 13;26(20):9954. doi: 10.3390/ijms26209954 (PMC12562319; doi:10.3390/ijms26209954)
Supplement: Supplementary file 1 [file ijms-26-09954-s001.zip › ijms-3909458-supplementary.pdf]

Supplementary Data

Supplementary Table S1: Calibration of telemetry device temperatures.

Temperature	Rectal core	Subcutaneous telemetry device	**Difference** (correction)
**7am**	37.1 ± 0.2°C	35.2 ± 0.3°C	1.9 ± 0.1°C
**3pm**	37.9 ± 0.2°C	35.8 ± 0.3°C	2.1 ± 0.1°C
**Average**			**2.1°C***

* Temperature correction, telemetry; n=6 mice

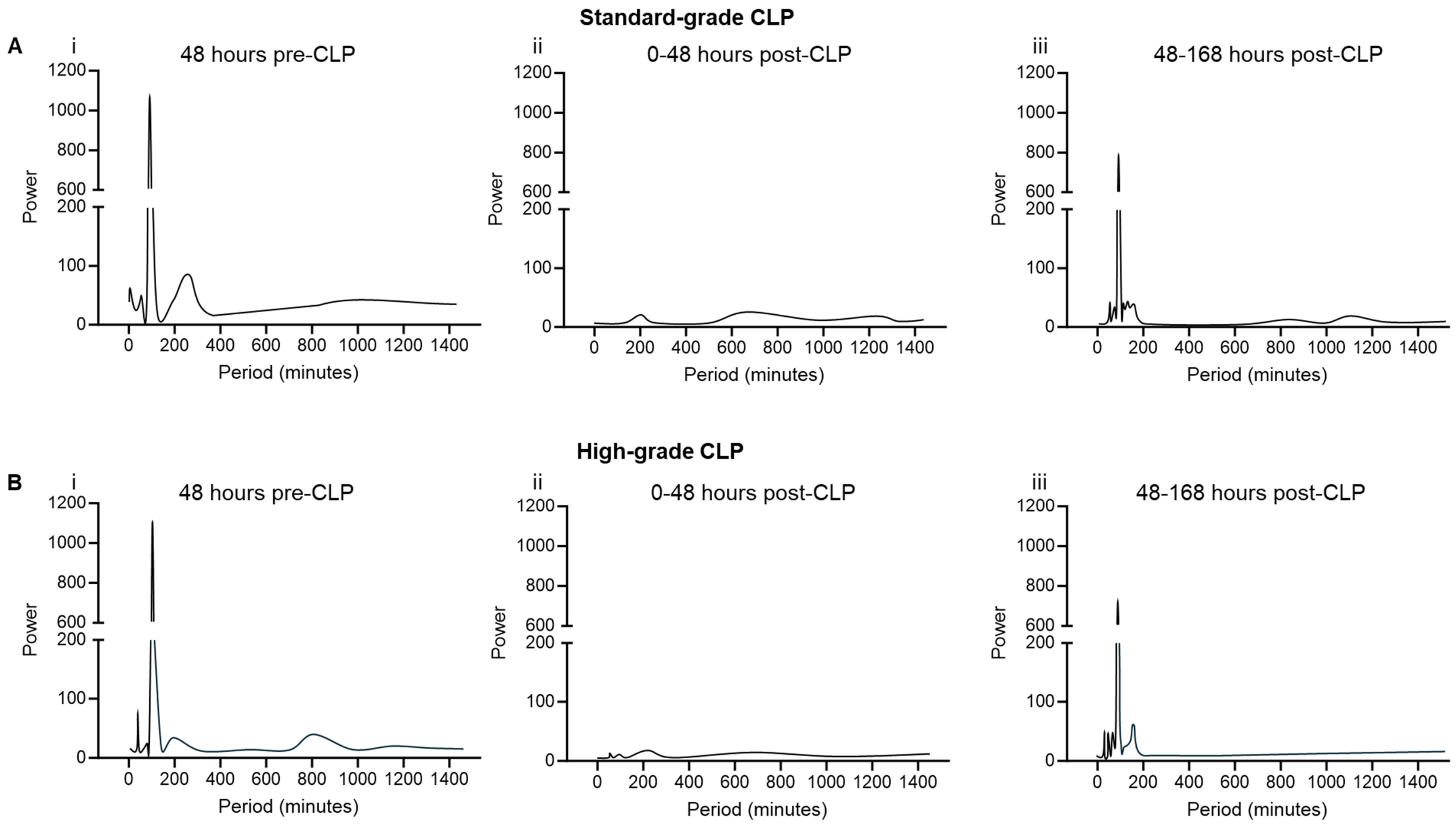

Supplementary Figure S1: Illustration of mean arterial pressure rhythms. Periodograms of mean arterial pressure from **(A)** standard-grade and **(B)** high-grade telemetry implanted mice in the **(i)** 48 hours prior to caecal ligation and puncture (CLP), **(ii)** 48 hours after CLP, and **(iii)** 48 to 168 hours after CLP.

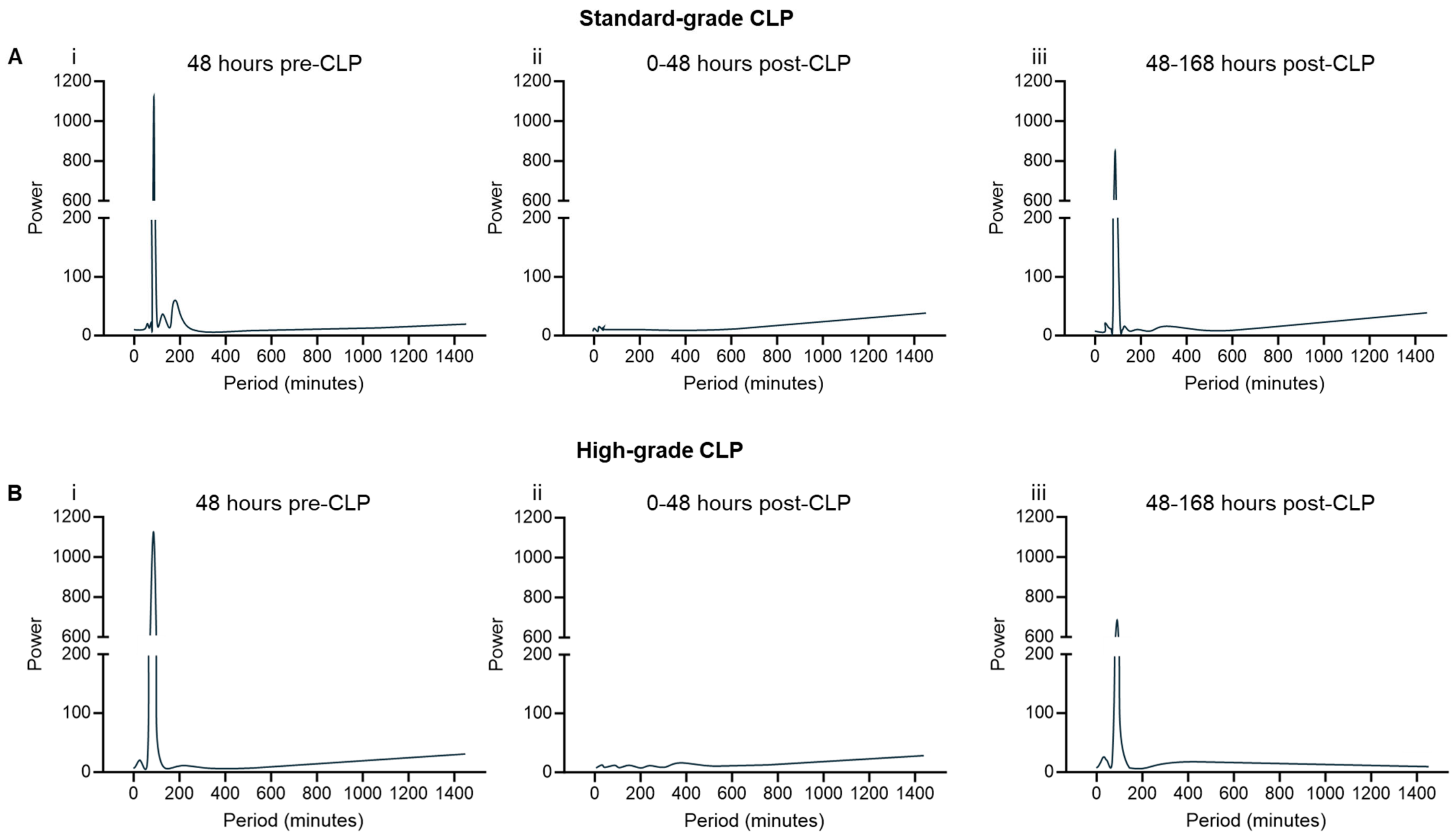

Supplementary Figure S2: Illustration of heart rate rhythms. Periodograms of heart rate from **(A)** standard-grade and **(B)** high-grade telemetry implanted mice in the **(i)** 48 hours prior to caecal ligation and puncture (CLP), **(ii)** 48 hours after CLP, and **(iii)** 48 to 168 hours after CLP.

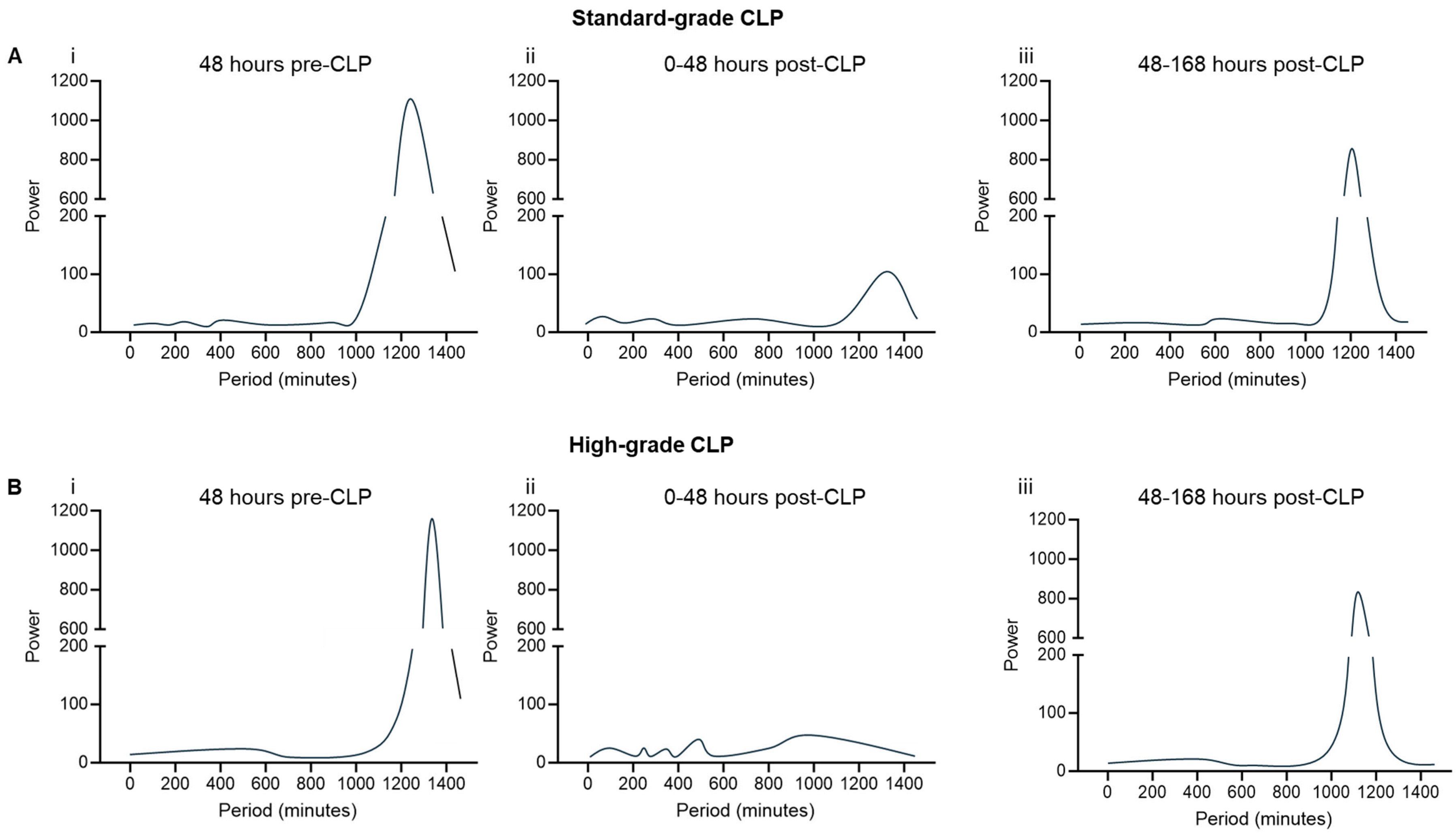

Supplementary Figure S3: Illustration of body temperature rhythms. Periodograms of temperature from **(A)** standard-grade and **(B)** high-grade telemetry implanted mice in the **(i)** 48 hours prior to caecal ligation and puncture (CLP), **(ii)** 48 hours after CLP, and **(iii)** 48 to 168 hours after CLP.
